# Alginate–Gelatin Hydrogel Scaffold Model for Hypoxia Induction in Glioblastoma Embedded Spheroids

**DOI:** 10.3390/gels11040263

**Published:** 2025-04-02

**Authors:** Janette del Rocío Aguilera-Marquez, Alejandro Manzanares-Guzmán, Lorena García-Uriostegui, Alejandro A. Canales-Aguirre, Tanya A. Camacho-Villegas, Pavel H. Lugo-Fabres

**Affiliations:** 1 Unidad de Biotecnología Médica y Farmacéutica, Centro de Investigación y Asistencia en Tecnología y Diseño del Estado de Jalisco (CIATEJ), Guadalajara 44270, Mexico; jaaguilera_al@ciatej.mx (J.d.R.A.-M.); almanzanares_al@ciatej.edu.mx (A.M.-G.); acanales@ciatej.mx (A.A.C.-A.); tcamacho@ciatej.mx (T.A.C.-V.); 2 SECIHTI-Secretaría de Ciencia, Humanidades, Tecnología e Innovación-Centro Universitario de Ciencias Exactas e Ingenierías, Universidad de Guadalajara, Guadalajara 44430, Mexico; lgarciaur@secihti.mx; 3 SECIHTI-Secretaría de Ciencia, Humanidades, Tecnología e Innovación-Unidad de Biotecnología Médica y Farmacéutica, Centro de Investigación y Asistencia en Tecnología y Diseño del Estado de Jalisco (CIATEJ), Guadalajara 44270, Mexico

**Keywords:** glioblastoma, 3D culture, scaffold-based spheroid model, hypoxia, HIF-1 α

## Abstract

Glioblastoma (GBM) is a highly aggressive and malignant brain tumor, characterized by hypoxia in its microenvironment, which drives its growth and resistance to treatments. Hypoxia-inducible factor 1 (HIF-1) plays a central role in GBM progression by regulating cellular adaptation to low oxygen availability, promoting processes such as angiogenesis and cell invasion. However, studying and modeling GBM under hypoxic conditions is complex, especially due to the limitations of animal models. In this study, we developed a glioma spheroid model using an alginate–gelatin hydrogel scaffold, which enabled the simulation of hypoxic conditions within the tumor. The scaffold-based model demonstrated high reproducibility, facilitating the analysis of HIF-1α expression, a key protein in the hypoxic response of GBM. Furthermore, cell viability, the microstructural features of the encapsulated spheroids, and the water absorption rate of the hydrogel were assessed. Our findings validate the three-dimensional (3D) glioblastoma spheroids model as a valuable platform for studying hypoxia in GBM and evaluating new therapies. This approach could offer a more accessible and specific alternative for studying the tumor microenvironment and therapeutic resistance in GBM.

## 1. Introduction

Glioblastoma (GBM), a highly aggressive and malignant primary brain tumor, is classified as a grade IV astrocytoma by the World Health Organization (WHO) [1]. Multimodal treatment involves maximal safe surgical resection, chemotherapy, and radiotherapy. Nevertheless, GBM cases still experience tumor progression and poor prognosis for patients [2,3]. Hypoxia is a distinctive feature of GBM, confirmed by necrosis or vascular proliferation within GBM tumors [4]. Despite being highly vascularized, GBM microcirculation is deficient, leading to highly hypoxic areas and necrosis within the tumor. As hypoxia gives rise to an inadequate nutrient supply and induces genetic changes, it is a major driver in tumor growth and plays a key role in a GBM patient’s prognosis [4]. GBM tumor cells can adapt and increase aggressiveness in response to low oxygen supply with cell proliferation augmentation in the surrounding anoxic zones [5,6]. Hypoxia gives rise to microvascular hyperplasia, an exacerbated angiogenesis that arises in response to the secretion of proangiogenic factors by the cells that form pseudopalisades as hypercellular zones neighboring a necrotic area.

Pseudopalisades are a distinctive feature of GBM histology. Thus, hypoxia-inducible factor 1 (HIF-1) is the master regulator in GBM aggressiveness via modulation of the cellular responses to hypoxia [7]. As such, HIF-1 is essentially related to GBM pathophysiology with pleiotropic effects on immunosuppression, angiogenesis, cell invasion, and survival under hypoxic conditions [8,9]. HIF-1 is a transcription factor comprised by subunits α and β, which have a similar basic helix–loop–helix and a Per/ARNT/Sim (PAS) domain conformation, but they differ in their transcriptional regulation mediated by oxygen [4]. Subunit β, also termed as aryl hydrocarbon receptor nuclear translocator (ARNT), is not reliant on oxygen. On the other hand, the α subunit is tightly regulated by oxygen. Thus, HIF-1α is rapidly degraded when oxygen is present. However, under hypoxic conditions HIF-1α is stable and translocates to the nucleus and heterodimerizes with HIF-1β, culminating in HIF-1 complex formation [4]. To bind to the hypoxia-response element (HRE) and subsequent coordination of the transcriptional response to hypoxia, HIF-1 needs cofactors, including p300/CREB-binding protein (CBP). Once HIF-1 reaches the nucleus, it prompts the activation of several genes involved in cell invasion, autophagy, angiogenesis, and lipid and glucose metabolism [8,10,11]. HIF-1 plays a driving role in angiogenesis through the transcription of several genes involved in abnormal vessel network establishment and metabolic reprogramming by activation of anaerobic and aerobic glycolysis, further accelerating tumor growth [4].

Notably, HIF-1α has been presumed to drive glioma progression from low-grade to GBM, as mRNA and protein expression have been linked to a higher pathological tumor grade and poor prognosis [4]. Tumor progression, higher pathological grade in brain tumors, and treatment resistance have been associated with HIF-1 and its gene targets, as a worse prognosis was related to their expression [4,9,12,13,14,15]. Correspondingly, a meta-analysis revealed that a higher tumor grade and worse overall survival rates observed in glioblastoma patients are connected to HIF-1 [4,16]. HIF-1α expression varies among glioblastoma grade, while HIF-1α expression is mainly located in the cytosol in low-grade gliomas (slow growing), contrasting to high-grade (faster growing), where HIF-1α is distinctively strong in the neighboring areas of necrosis, mostly found in nuclei [4,13,17].

Moreover, HIF-1 is controlled by various mechanisms, including through the activation of oncogenes like EGFR (epidermal growth factor receptor), or inactivation of tumor suppressor genes, including p53, phosphatase, and the tensin homolog (PTEN) [4,5]. EGFR amplification is present in more than 50% of GBM cases, portraying one of the prevalent molecular characteristics. Therefore, HIF-1α transcription can be activated by the MAPK/ERK signaling pathway/signal transducer and activator of transcription 3 (STAT3). Furthermore, HIF-1α is also recognized as a downstream target of the oncogenic pathway PI3K/AKT/mammalian target of rapamycin (mTOR) [4].

Hypoxic conditions within the tumor favor its progression, leading to chemoresistance and radioresistance. Radiation therapy, such as ionizing radiation, is feeble upon oxygen deprivation. Since ionizing radiation comprises direct damage to DNA molecules and subsequent generation of free radicals, including reactive oxygen species (ROS), and oxygen is necessary to stabilize the DNA strand breaks caused by radiotherapy, the reduced levels of oxygen within the tumor induce treatment resistance [18,19,20,21]. HIF-1 impairs ROS production via PDK1 activation, raising an antioxidant system, followed by ROS levels decline, and culminating in radioresistance. Moreover, the inhibition of HIF-1α sensitizes glioma cells to temozolomide, as reported in HIF-1α knocked-down cell models [22]. Therefore, hypoxia and HIF-1α must be considered fundamental aspects and studied in detail to elucidate their role in the onset and progression of GBM. Nevertheless, several variables in vivo are intractable, encompassed by unknown effects owing to the complexity of organisms [23], translating into therapies that fail during clinical trials due to insufficient models that properly predict the efficacy and toxicity of such therapies in humans [24]. Several aspects of GBM are influenced by the tumor microenvironment (TME), such as metastasis, immune cell modulation, cytotoxicity resistance, angiogenesis, and tumor development [25,26]. Thus, there is a pressing need for more accessible GBM preclinical models that enable better control of variables through a reductionist approach that accurately models a specific feature of the disease, as opposed to animal models, which not only have ethical issues, but are uncontainable for a specific aspect of GBM, owing to the complexity of the in vivo systems, and also differ from human disease [27]. The 3D cell culture is adequate for succeeding in these objectives by granting more consistent models capable of restraining a specific feature of GBM by selectively using reductionist models, leading to the improved measurement of outcomes in a controlled manner and allowing the correlation between the TME, tumor recurrence, and even therapy resistance [23,24].

The 3D cell culture encompasses comprehensive cell culture techniques to grow cells in three dimensions across an artificial and accurate environment. Notably, the 3D culture exhibits physiological cell–cell and cell–ECM (extracellular matrix) component interactions, granting the growing cells in a TME with the capability to accurately capture GBM in vivo conditions [25,27,28,29]. In addition, the ECM can interfere with the mechanism of action of a given drug, through alteration of the cell responses toward the former, culminating in an increase of drug resistance, or its therapeutic effectiveness [24]. A 3D model emulates the TME of the tissue, granting the cells with the means to proliferate, differentiate, and gather, which could, in turn, contribute to a more precise estimate of drug efficacy within a cell [30]. In addition, receptor tyrosine kinases, including EGFR and integrins, can interact with ECM components, as such, the crosstalk between them regulates downstream cell signaling and EGF-induced biological activity, which translates into cell proliferation and invasion [24,31].

Therefore, 3D culture enables resemblance of in vivo GBM tumor microenvironment through cell–ECM component interactions. Moreover, biological/natural scaffolds supply backing for cell growth and comparable in vivo microenvironment with ECM components, growth factors, and hormones. Biological scaffolds can also be controlled for similar in vivo composition, elasticity, and porosity to improve the ECM appearance. Biocompatibility, spatial distribution, and lower toxicity can also be improved within biological scaffolds. The biological scaffolds can comprise ECM components, including agarose, collagen, laminin, fibroin, chitosan, hyaluronic acid, starch, human decellularized ECM, alginate, and gelatin [32,33,34,35]. Notably, cellular functions can be influenced by the microscale mechanical features of biomaterials, including structural integrity, interconnectivity, stiffness, and porosity [36,37].

Due to the disparate vascular supply, cells in the 3D spheroids have variable conditions, namely limited nutrients, waste removal, and oxygenation. The 3D spheroids can have an oxygen-deprived core (hypoxic core) like those TMEs found in solid tumors, holding cells at the center of the sphere with low oxygen, glucose concentration, and acidic extracellular pH owing to the accumulation of metabolic by-products [38,39]. Remarkably, the increase in hypoxic cell population is proportional to the spheroid size, and the former is highly resistant to chemotherapy and radiotherapy. Whereas viable proliferating cells are found in the outer layer of the spheroid, which is also highly exposed to the medium [40]. As such, the heterogenous cellular subpopulation within the 3D spheroid, including the actively proliferating, quiescent, hypoxic, and necrotic cells, offers distinctive cell proliferation zones, namely the proliferating zone, the quiescent viable zone, and the necrotic/hypoxic core [28,31,33]. Several aspects of the 3D spheroids affect cellular function, including gene expression, communication, morphology, gene expression, responses to external stimuli, differentiation, proliferation, and survival, which are influenced by the cellular organization, polarity, and geometry of the 3D spheroids [24,30]. Nevertheless, the drawbacks of the currently existing 3D cell culture are that it could be expensive, time consuming, have restricted intratumoral heterogeneity, and may have lower reproducibility [41]. Thus, new developments to validate automation and high throughput analyses to ensure the reproducibility of 3D cell culture models are imperative [42].

In the present study, we successfully developed a cost-effective, highly reproducible three-dimensional (3D) alginate–gelatin scaffold-based glioblastoma spheroid model of the U87-MG cells, which has the capacity to induce a hypoxic microenvironment through HIF-1α, resembling the heterogeneous population of actively proliferating, hypoxic, and necrotic cells found in the TMEs of GBM tumors. We evaluated the alginate–gelatin hydrogel scaffold reproducibility, characterized it by ATR-FTIR spectroscopy, assessed its kinetics of water absorption, generated homogenous U87-MG spheroids in terms of size and reproducibility, evaluated the cell viability of these embedded spheroids within the alginate–gelatin hydrogel scaffold, determined the microstructural features of the encapsulated U87-MG spheroids in the hydrogel scaffolds using a scanning electron microscope (SEM), and examined the encapsulated U87-MG spheroids in the hydrogel scaffolds through confocal microscopy and detected HIF-1α expressed within the 3D alginate–gelatin scaffold-based glioma spheroid model with an anti-HIF-1α mouse monoclonal antibody. Our study provides a 3D alginate–gelatin scaffold-based glioblastoma spheroid model and experimental basis for developing a cost-effective and highly reproducible model to induce hypoxia, a distinctive feature, and one of the major drivers in the progression of GBM tumors. Our model could contribute to further elucidating the role of HIF-1α and evaluating the efficacy of the forthcoming therapies intended for glioblastoma.

## 2. Results and Discussion

### 2.1. The Alginate–Gelatin Hydrogel Scaffold Demonstrated Suitable Reproducibility

**Figure 1:**
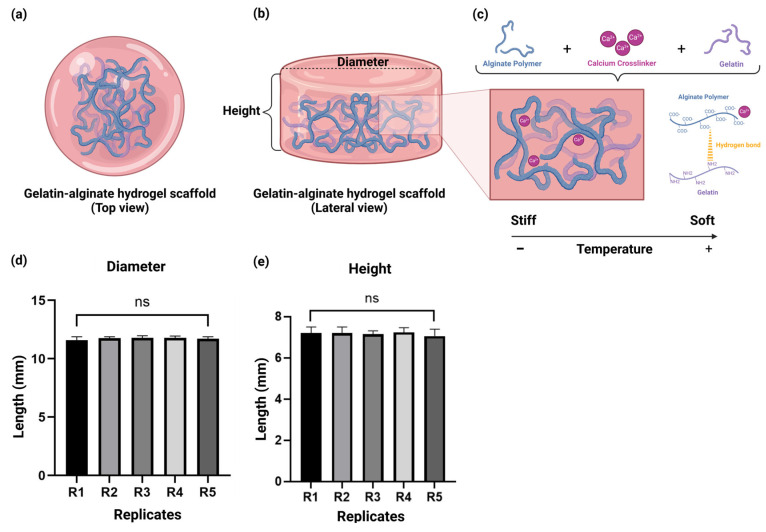
The alginate–gelatin hydrogel scaffold. (**a**) Schematic representation of the alginate– hydrogel scaffold (Top view). (**b**) Schematic representation of the alginate–gelatin hydrogel scaffold (Lateral view). (**c**) Schematic representation of the molecular crosslinking of the alginate–gelatin hydrogel scaffold. Evaluation of the manufacturing reproducibility of the alginate–gelatin hydrogel scaffolds of the parameters: (**d**) diameter, and (**e**) height. Data is presented as a mean ± SD. The experiments were performed in quintuples to demonstrate the reliability of our measurements. Student’s *t*-test for the comparison amongst the alginate–gelatin scaffold replicates (*n* = 5), considering *p* < 0.05 significant, ns > 0.05. R = Replicate.

**Figure 2:**
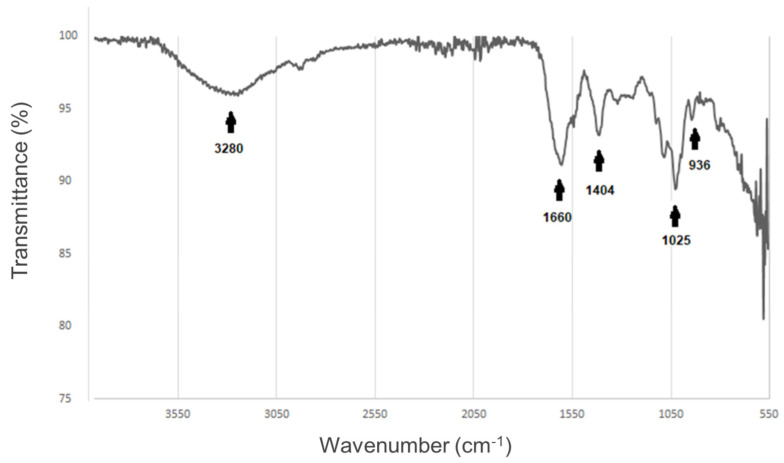
Fourier transform infrared spectrum of the alginate–gelatin hydrogel scaffold. The infrared spectrum of the scaffold exhibits distinctive peaks for the different functional groups within the sample (black arrows). From left to right, O-H group at 3280 cm^−^^1^, –CONH_2_ group occurs at 1660 cm^−^^1^, C-OH at 1404 cm^−^^1^, CH group 1025 cm^−^^1^ corresponding to the glucuronic units, CH group of the pyranose ring at 936 cm^−^^1^. The functional groups were added in BioRender (https://www.biorender.com/).

As depicted, the schematic representation portrays the interaction amongst the alginate–gelatin (Figure 1a,b), and their molecular crosslinking in the hydrogel scaffold (Figure 1c) after Ca^2+^ addition as a crosslinker favoring hydrogel polymerization. Moreover, this was further confirmed by the chemical characterization with ATR-FTIR (Figure 2). To validate the manufacturing reproducibility of our alginate–gelatin scaffold, we performed the following experiments. Hence, we evaluated two parameters: diameter and height. As depicted in Figure 1d,e, the results showed a diameter of 11.7 mm ± 0.2 and a height of 7.2 mm ± 0.3. Moreover, no significant differences were observed between the replicates (*n* = 5). Thus, the reproducibility of the scaffold properties was consistent and maintained amongst the replicates.

Glioblastoma (GBM) is a highly aggressive and malignant primary brain tumor. GBM cases still experience tumor progression and poor prognosis for patients [2,3]. Hypoxia is a distinctive feature of GBM, which is confirmed by necrosis or vascular proliferation within GBM tumors [4]. Hypoxia contributes to the formation of microvascular hyperplasia, an exacerbated angiogenic form that leads to the secretion of proangiogenic factors by the cells of the hypercellular zones neighboring a necrotic area, specifically the pseudopalisades, which are a distinctive feature of GBM histology. Furthermore, hypoxic conditions within the tumor favor its progression, leading to chemoresistance and radioresistance [4]. Hence, hypoxia-inducible factor 1 (HIF-1) is the master regulator in GBM aggressiveness via modulation of cellular responses to hypoxia [7]. HIF-1α has been presumed to be a major driver of glioma progression from low-grade to GBM [4]. Consistently, the inhibition of HIF-1α sensitizes glioma cells to temozolomide, as reported in HIF-1α knocked-down cell models [22]. Elucidation of hypoxia and HIF-1α role in the progression of GBM are imperative to hold out against the corresponding chemoresistance and radioresistance linked to these factors. Therefore, there is a crucial requirement for accessible GBM preclinical models that enable better control of variables through reductionist and consistent methods that reliably model a specific hallmark of the disease, such as hypoxia. Nevertheless, this is hardly feasible in the in vivo systems, due to the complexity of the in vivo system, which limits the ability to control a specific feature of GBM. Furthermore, there is a divergence of animal models from the human disease [23], which translate into therapies that fail during clinical trials due to insufficient models that properly predict the efficacy and toxicity of such therapies in humans [24]. A 3D cell culture is a suitable choice to overcome these drawbacks, by providing a more selective, consistent, and reductionist model capable of dissecting a specific feature of GBM, such as hypoxic conditions related to HIF-1α. A 3D cell culture has physiological cell–cell and cell–ECM component interactions, which allow cells to grow in a TME that closely resembles GBM in vivo conditions [24,25,28]. However, the downside of the current 3D cell culture is that it can be time-consuming, expensive, and has restricted reproducibility [41,43]. Thus, there is an imperative need to develop replicable 3D cell culture models that consistently accomplish high throughput analyses, validate automation, and proficiently emulate a specific feature of GBM. We successfully established a cost-effective and reproducible method for the 3D cell culture of U87-MG spheroids encapsulated in an alginate–gelatin hydrogel scaffold model, which induced hypoxia through HIF-1α expression. We rigorously developed this model as a systematic procedure.

We characterized the physicochemical characteristics of our alginate–gelatin hydrogel scaffold as follows. First, we evaluated the manufacturing reproducibility of the alginate–gelatin hydrogel scaffold by the assessment of diameter and height, which showed a diameter of 11.7 mm ± 0.2 and a height of 7.2 mm ± 0.3 (Figure 1d,e). We then successfully demonstrated the reproducibility and consistency of the scaffold properties, as no significant differences were observed between the replicates (*n* = 5).

### 2.2. ATR-FTIR Spectroscopy Suggest That the Negative Group of the Alginate Could Be Associated with the Positive Charge of the Gelatin

To detect the changes in the chemical structure, owing to the crosslinking amongst gelatin and alginate, we performed a characterization of our alginate–gelatin hydrogel scaffold via ATR-FTIR spectroscopy. The ATR-FTIR spectroscopy measures the frequency of the absorption bands and changes in the relative intensities of the bands. As seen in Figure 2 a distinctive amide group (-CONH_2_ group) signal was detected, suggesting an interaction between gelatin and alginate, this peak is observed at 1660 cm^−^^1^. Thus, this finding implies that the positive charge of the gelatin (NH_2_) could be linked with the negative group of alginates (COO^−^), as depicted in Figure 1c.

The ATR-FTIR spectroscopy analysis revealed a distinctive amide group (–CONH_2_ group) signal, suggesting an interaction between gelatin and alginate. This peak is observed at 1660 cm^−1^ (Figure 2). This result implied that the positive charge of the gelatin (NH_2_) could be linked with the negative group of alginates (COO^−^) (Figure 1c). Hydrogels, a type of hydrophilic polymer, are increasingly used to create 3D environments for tumor cells due to their biocompatibility and similarity to the natural extracellular matrix (ECM) [44]. These materials support cell growth, migration, and survival, making them valuable alternatives to traditional 2D models in cancer research, particularly for testing and screening anti-cancer drugs [44,45]. Research shows that hydrogel systems better replicate the interactions between cells and the matrix in tumors, promoting cell aggregation and spheroid formation [46,47]. Natural polymers, like sodium alginate (SA), are especially useful for hydrogel creation, given their biocompatibility and biodegradability. These hydrogels can be cross-linked using multivalent cations like Ca^2+^ [48] and are widely used for 3D cancer cell culture and drug delivery [44,49,50,51]. However, to more accurately mimic the native ECM, protein components, such as gelatin, a derivative of collagen, are also added [44,52]. Gelatin is biocompatible, biodegradable, and cost-effective, and when combined with alginate, it enhances cell adhesion, migration, and proliferation, making the hydrogel suitable for cell encapsulation [53,54,55]. Moreover, gelatin contains the RGD sequence for cell adhesion and ensures mechanical support to the final matrix [56].

Gelatin and alginate are natural polymers that offer distinct benefits as scaffolds for glioblastoma research. Gelatin, which is obtained from animal connective tissues, is a thermoplastic substance that transforms into a soft hydrogel at ambient temperatures. This property makes it highly effective for crafting tailored scaffolds through molding, while also facilitating the attachment and growth of glioblastoma cells due to its ability to promote cell adhesion. Gelatin contains amino acid sequences, such as –RGD and –GFOGER, which emulate the extracellular matrix (ECM), creating a more accurate environment for tumor cells [57,58]. Its biocompatibility ensures it does not provoke substantial inflammatory responses, an essential feature for long-term experiments. Alginate, a polysaccharide sourced from brown algae, is another biocompatible polymer that forms semi-transparent hydrogels when exposed to calcium ions [59,60]. Although it does not support cell adhesion as effectively as gelatin, alginate’s capacity to form stable, calcium-dependent gels enables the controlled release of bioactive substances, which can be beneficial in modulating the tumor microenvironment. This characteristic makes it especially useful for glioblastoma research, where understanding tumor growth, cell movement, and therapeutic resistance is key. Despite its limitations in cell adhesion, the combination of gelatin and alginate overcomes these issues by harnessing the advantages of both materials. The use of both gelatin and alginate in a single hydrogel scaffold presents a novel method for simulating glioblastoma. This mixed hydrogel can be easily shaped via thermoplastic molding, followed by immersion in a calcium solution, which streamlines the fabrication process and ensures sterilization [61]. Autoclaving further guarantees the scaffold’s stability and biocompatibility. This approach allows scaffolds to be molded into various forms [61], offering flexibility in experimental setups for studying glioblastoma cells in three-dimensional environments. By combining the properties of both gelatin and alginate, researchers can create more physiologically relevant models of glioblastoma, which closely replicate the tumor microenvironment. These scaffolds support the growth and movement of cells, making them ideal for exploring glioblastoma’s complex behavior, assessing drug responses, and investigating potential therapies. The ease with which scaffolds can be customized, coupled with the biocompatibility of the materials, makes them valuable tools in cancer research and treatment development.

### 2.3. Kinetics of Solvent Absorption in the Alginate–Gelatin Hydrogel Scaffold

**Figure 3:**
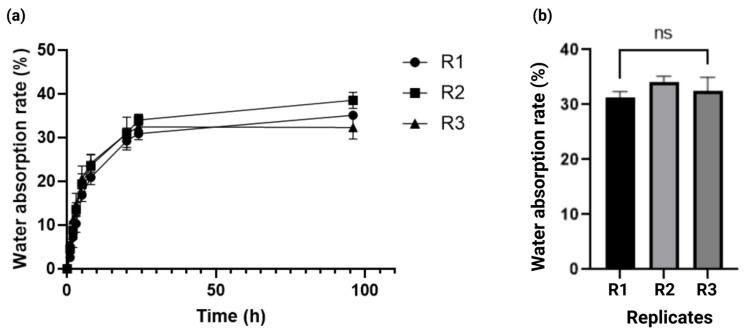
Kinetics of water absorption rate of the alginate–gelatin hydrogel scaffold. (**a**) Water absorption rate in the alginate–gelatin hydrogel scaffold for 96 h. Data is presented as a mean ± SD. The experiments were performed in triplicate. (**b**) One-way ANOVA and Tukey’s post hoc pairwise comparison of water absorption rate of the alginate–gelatin hydrogel scaffold between the replicates after 96 h of incubation, considering *p* < 0.05 significant, ns > 0.05. R = Replicate.

To determine the kinetics of solvent absorption in the alginate–gelatin hydrogel scaffold, we measured the water absorption rate of the alginate–gelatin hydrogel scaffold weight as a function of time, for up to 96 h. As observed in Figure 3a, the maximum percentage of water absorption rate was 32.5% ± 2 at 24 h. Furthermore, the water absorption rate had a variation of less than 5% from 24 to 96 h, demonstrating solute diffusion stability within the alginate–gelatin hydrogel scaffold. Notably, these results were consistent between the replicates. The kinetics of the water absorption rate demonstrated solute diffusion stability within the alginate–gelatin hydrogel scaffold and consistency amongst the replicates (Figure 3a,b).

### 2.4. Spheroid Size Demonstrated Reproducible and Homogenous Size Amongst Replicates

**Figure 4:**
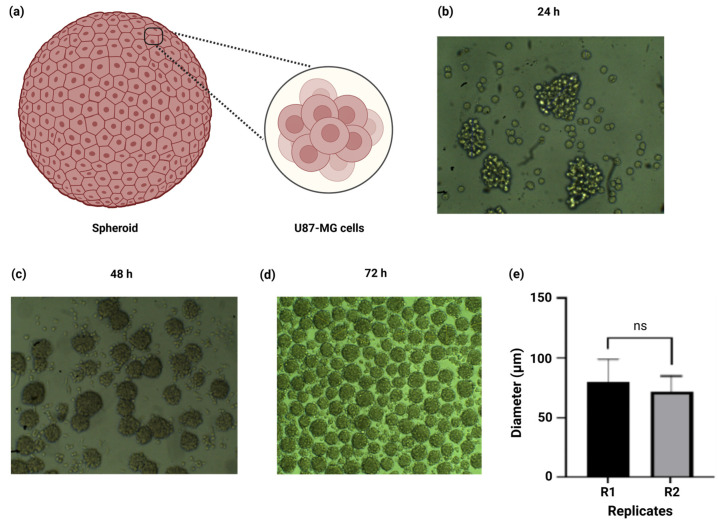
Development of spheroids from a U87-MG cell line in low-adherence plates. (**a**) Schematic representation of spheroid cell composition. (**b**) The U87-MG cell culture and subsequent spheroid formation after 24 h (10× magnification). (**c**) Spheroids of variable sizes after 48 h incubation (10× magnification). Numerous spheroids of homogenous size are observed after 72 h incubation (4× magnification). (**d**,**e**) Determination of spheroid diameter and comparison amongst the replicates. Data is presented as a mean ± SD. The experiments were performed in triplicate (*n* = 3 per replicate). Post hoc Student’s *t*-test for comparison of the replicates, considering *p* < 0.05 significant, ns > 0.05.

After the physicochemical characterization of the alginate–gelatin hydrogel scaffold, we proceeded with the development of spheroids from a U87-MG cell line. We successfully refined the spheroid formation procedure through cell cultures in low-adherence plates without medium removal, which allowed constant agitation and self-aggregation of the cells for 3 days. Further measurement of the spheroid’s diameter of 100 mm determination of the cell number within the spheroids was performed by light microscopy. A schematic representation of the U87-MG spheroid composition is depicted in Figure 4a. The U87-MG cell aggregation and initiation of spheroid formation arises after 24 h of incubation with variable spheroid sizes (Figure 4b). Our results indicate that the homogenous size and quantity of the spheroids increased in a time-dependent manner, from 48 h (Figure 4c) to 72 h (Figure 4d). Moreover, we determined the number of spheroids formed within each well, obtaining 930 ± 60 spheroids/well (Figure 4d). The diameter of the spheroids obtained was 75.7 ± 17 µm, and there were no statistically significant differences between the replicates (Figure 4e). Based on the cell count, each spheroid comprised 1089 ± 282 U87-MG cells.

We successfully refined the spheroid formation procedure through cell culture in low-adherence plates without medium removal, which allowed constant agitation and self-aggregation of the cells for 3 days. Further measurement of the spheroid’s diameter and determination of the cell number within the spheroids was performed by light microscopy (Figure 4b–d). One study reported a similar approach with a gelatin–alginate hydrogel for the 3D culture of embedded glioma cells and U87-MG spheroids; however, despite a detailed characterization, neither the diameter of the spheroids, the number of spheroids/well, nor the cell count per spheroid were disclosed [44]. Furthermore, the U87-MG spheroids (10^3^ U87-MG cells) with a diameter of 260 µm to 290 µm after 3 days of incubation have been reported [62]. These apparent inconsistencies could be attributed to the difference in the hanging drop method and the fact that, as we mentioned earlier, the number of spheroids formed within each well increased in a time-dependent manner, translating into smaller spheroids. Despite the simplicity and low cost of the hanging drop method, one drawback is its susceptibility to droplet detachment, translating into complications for high-throughput screening and the inclusion of stromal cells to mimic the TME, especially when adding liquids like drugs or stromal/immune cells directly to the spheroid-containing drop [63]. Nevertheless, in our study the diameter size of the spheroids was consistent amongst the replicates, further confirming the reproducibility and homogeneous size distribution of our U87-MG spheroids.

### 2.5. Cell Viability Assays of the Spheroids Within the Scaffolds

**Figure 5:**
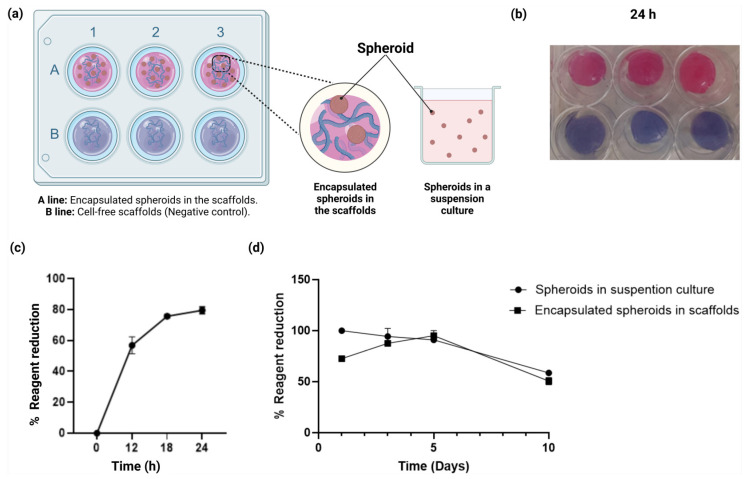
Cell viability assays of the U87-MG spheroids embedded in the alginate–gelatin hydrogel scaffold. (**a**) Schematic representation of the cell viability assays of the U87-MG spheroids embedded in the scaffolds (upper and lateral view of the scaffold wells) and spheroids in a suspension culture. (**b**) Cell viability assays of the encapsulated spheroids in the scaffolds. Encapsulated U87-MG spheroids in the scaffolds (top) and cell-free scaffolds (bottom) were incubated for 24 h with alamarBlue™ solution. Cell-free scaffolds were used as negative control. The experiments were performed in triplicate. (**c**) Cell viability assays of the encapsulated spheroids in the scaffolds, evaluation of the cellular metabolic activity as a function of the resazurin reagent reduction (%) for 12, 18, and 24 h. The means for each concentration are represented. The experiments were performed in triplicate. (**d**) Cell viability assays of the encapsulated spheroids in the scaffolds and spheroids in a suspension culture, evaluation of the cellular metabolic activity as a function of the resazurin reagent reduction (%) for 1, 3, 5, and 10 days. The means for each concentration are represented. The experiments were performed in triplicate. The post hoc Student’s *t*-test for independent samples considering *p* < 0.05 significant, showed no significant difference in cell viability amongst encapsulated spheroids and spheroids in a suspension culture.

To evaluate the biocompatibility of the alginate–gelatin hydrogel scaffold with the U87-MG cells, we carried out a cell survival test of the encapsulated spheroids in the scaffold (Figure 5a). Simultaneously, we also determined the optimal incubation time of alamarBlue™ solution, based on the measurement of the metabolic capacity of living cells through the reduction of oxidized non-fluorescent blue resazurin to a red fluorescent dye (resorufin) by the active mitochondrial respiratory chain in living cells, according to the manufacturer’s recommendations. Owing to the non-toxic properties of resazurin, we were able to evaluate the encapsulated spheroids in the scaffolds at 0, 12, 18, and 24 h (Figure 5b).

Based on these results, we determined that the optimal incubation time of the encapsulated spheroids in the scaffold was 24 h, with the resazurin maximum reduction of 79.5% ± 2.2. Accordingly, the incubation time of the encapsulated spheroids in the scaffolds coincided with a maximum percentage of the water absorption rate, as the latter is required for the diffusion of the reagent within the scaffold. Next, we assessed the cell viability of the U87-MG spheroids embedded in the alginate–gelatin hydrogel scaffold and compared them with the cell-free alginate–gelatin hydrogel scaffold (negative control) for 24 h (Figure 5c). The alginate–gelatin hydrogel scaffold demonstrated suitable diffusion of the reagent in both groups. Nevertheless, only the encapsulated spheroids showed a reduction in the alamarBlue™ solution (1×), owing to the metabolic activity of the U87-MG cells within the spheroids.

Finally, we evaluated the cell viability of the encapsulated U87-MG cell spheroids compared to the U87-MG spheroids in a suspension culture for up to 10 days (Figure 5d). The alginate–gelatin hydrogel scaffold showed suitable biocompatibility in the encapsulated spheroid group (Figure 5d). As seen, the U87-MG spheroids in a suspension culture and the encapsulated spheroid group exhibited different growth patterns. Suspension culture initiated in 95% ± 5 on Day 1, but showed a decrease in the cellular metabolic activity in a time-dependent manner, unlike the 3D encapsulated U87-MG cell spheroids group. However, there seems to be an apparent lower cellular metabolic activity from Day 1 in the encapsulated U87-MG cell spheroids group, when compared to the U87-MG spheroids in a suspension culture group. This effect is associated with the crosslinking process of the scaffolds, as the encapsulated spheroids within the alginate–gelatin hydrogel scaffolds are refrigerated for the subsequent crosslinking process. Nevertheless, we observed an increase in reduction activity in a time-dependent manner, showing a 95% ± 5 increase on Day 5, which could imply the adaptation of the U87-MG spheroids to the alginate–gelatin hydrogel scaffolds. From Day 5 forward, a decrease in cellular metabolic activity is observed for both groups (Figure 5d).

As mentioned earlier, to evaluate the biocompatibility of the alginate–gelatin hydrogel scaffold with the U87-MG cells, we carried out a cell survival test for the encapsulated spheroids in the scaffold (Figure 5a), and determined the optimal incubation time of the alamarBlue™ solution, based on the measurement of the metabolic capacity of living cells through the reduction of oxidized non-fluorescent blue resazurin to a red fluorescent dye (resorufin) by the active mitochondrial respiratory chain in living cells, according to the manufacturer’s recommendations. Next, we were able to assess the encapsulated spheroids in the scaffolds at 0, 12, 18, and 24 h (Figure 5b). The optimal incubation time of the spheroids in the scaffold was 24 h, with the maximum reduction of 79.5% ± 2.2, which was also consistent with the maximum percentage of water absorption rate, a crucial requirement for diffusion of the reagent within the scaffold. For reliable cell adhesion and culture, the surface of the hydrogel must be smooth and uniform. If the surface is irregular or distorted, the hydrogel is not appropriate as a substrate for cell growth. Such distortions in the shape of the hydrogel may result from uneven shrinkage of the material or the separation of its components [61]. Consistently, our scaffolds were flat and smooth, which enabled the adherent culture of cells reproducibly (Figure 5b). The diffusion of the reagent was demonstrated in subsequent cell viability assays of the U87-MG spheroids in the alginate–gelatin hydrogel scaffold, compared to the cell-free alginate–gelatin hydrogel scaffold (negative control) for 24 h (Figure 5c). However, only the encapsulated spheroids showed reduction of the alamarBlue™ solution (1×), owing to the metabolic activity of the U87-MG cells within the spheroids. Finally, cell viability of the encapsulated U87-MG cell spheroids exhibited different growth patterns, when compared to a suspension culture for up to 10 days (Figure 5d). The suspension culture was initiated in 95% ± 5 on Day 1, but showed a decrease in the cellular metabolic activity in a time-dependent manner, unlike the 3D encapsulated U87-MG cell spheroids group. An increase in the reduction activity was observed in a time-dependent manner, showing a 95% ± 5 increase on Day 5, which could imply the adaptation of the U87-MG spheroids to the alginate–gelatin hydrogel scaffolds. From Day 5 forward, a decrease in cellular metabolic activity is observed for both groups (Figure 5d). Composition of the alginate–gelatin proportion within the hydrogel scaffold is a relevant feature. The cytocompatibility of the alginate–gelatin hydrogel has been reported in a 3D culture system in GBM cells with 5w% gelatin and 5w% sodium alginate [44]. Here, we report a 3.75w% alginate and 2.5w% gelatin with cell viability of 95% ± 5 on Day 5 for the encapsulated U87-MG spheroids in the alginate–gelatin hydrogel scaffold, which demonstrated that our alginate–gelatin hydrogel scaffold was biocompatible and suitable to sustain incubation of the encapsulated alginate–gelatin hydrogel scaffold for up to 10 days.

### 2.6. Microstructural Analyses with SEM and Cell Adhesion of U87-MG Cells to the Scaffold Structure

**Figure 6:**
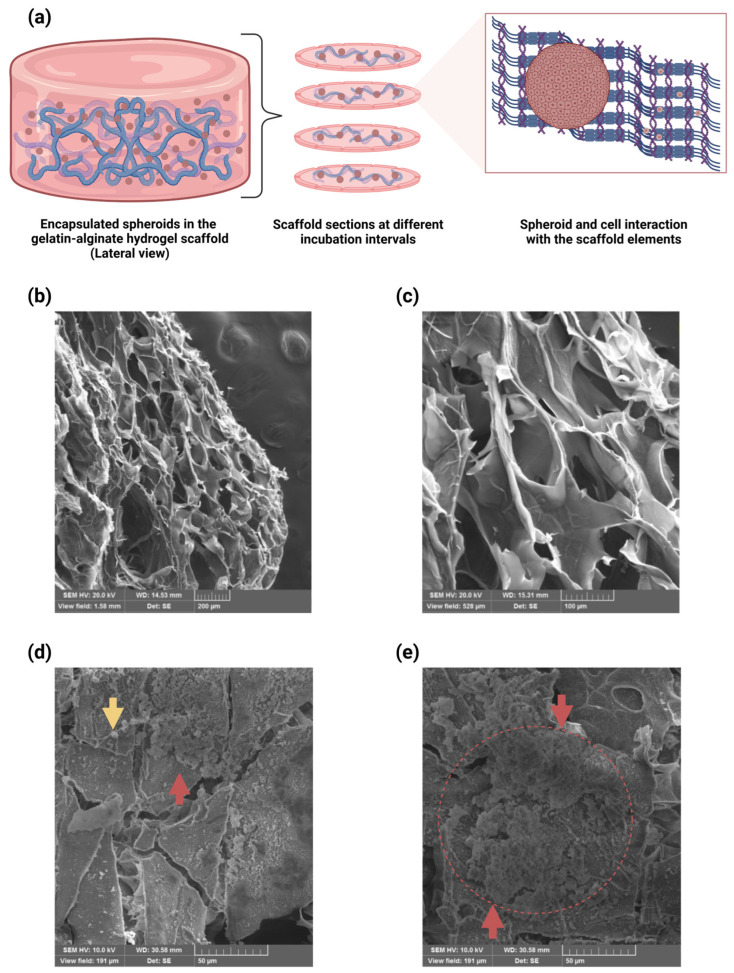
SEM micrographs of the encapsulated U87-MG spheroids in the alginate–gelatin hydrogel scaffold. (**a**) Schematic representation of the encapsulated spheroids in the scaffold. Longitudinal scaffold sections were generated with cryostat cutting, and encapsulated spheroid and cell interaction with the scaffold elements. (**b**,**c**) SEM micrograph of the alginate–gelatin hydrogel scaffold with the encapsulated spheroids. Microstructural analyses with SEM revealed heterogeneous pore size distribution, such as small pores embedded in larger ones, bearing interconnected adjacent pores. SEM magnifications: 121× (**b**) and 361× (**c**). (**d**,**e**) SEM micrograph of the encapsulated spheroid and cell adhesion to the alginate–gelatin hydrogel scaffold structures. The SEM micrographs show U87-MG cell adhesion to the alginate–gelatin hydrogel scaffold structures (yellow arrow) (**d**), or collapsed (**e**), the U87-MG spheroids in the alginate–gelatin hydrogel scaffold structures (red arrows and circle). SEM magnifications: 1.00k× (**d**,**e**). The experiments were performed in triplicate.

To determine the microstructural characteristics of the alginate–gelatin hydrogel scaffold and cell attachment to the scaffold, we characterized the morphology of the scaffolds with a scanning electron microscope (SEM). Figure 6a describes the process of analysis of the encapsulated spheroids. Subsequent analyses were performed using ImageJ software version 1.54 to measure the area of the pore cross sections. Microstructural analyses with SEM revealed heterogeneous pore size distribution, such as small pores embedded in larger ones, bearing interconnected adjacent pores (Figure 6b,c). The cross-sectional area of the pore sizes was 76–230 µm. These results imply that our alginate–gelatin hydrogel scaffold could represent an advantageous and cost-effective model for the assessment of antineoplastic drugs, as our scaffold micrometric attributes emulates relevant GBM tumor features. Moreover, the SEM micrographs show U87-MG cell adhesion to the alginate–gelatin hydrogel scaffold structures (Figure 6d) and the collapsed U87-MG spheroids in the alginate–gelatin hydrogel scaffold structures (Figure 6e). 

A 3D culture enables the resemblance of an in vivo GBM tumor microenvironment through cell–ECM (extracellular matrix) component interactions. Moreover, biological/natural scaffolds supply the backing for cell growth and a comparable in vivo microenvironment with ECM components, growth factors, and hormones. Notably, the cellular function can be influenced by the microscale mechanical features of the biomaterials, including structural integrity, interconnectivity, stiffness, and porosity [24,36]. Determination of the microstructural features of the alginate–gelatin hydrogel scaffold and cell attachment to the scaffold was achieved via SEM analysis, which revealed suitable pore sizes for diffusion and cell adhesion of the U87-MG cells to the scaffold structure. Microstructural analyses with SEM revealed heterogeneous pore size distribution, such as small pores embedded in larger ones, bearing interconnected adjacent pores (Figure 6b). Our results are consistent with a previous study reporting a similar gelatin–alginate hydrogel scaffold and U87-MG cells attachment [44]. However, the cross-sectional area of the pores sizes was not disclosed. The cross-sectional area of the pore sizes of our alginate–gelatin hydrogel scaffold was 76–230 µm. Florczyk et al., (2013) reported scaffolds for human glioblastoma cell culture in vitro, showing cell growth, spheroid formation, and gene expression-like in vivo tumors, with an average pore size of 77 μm [64]. Tang et al., (2020) described a GBM model with a mean pore size of 85 μm, allowing for small drug molecules to diffuse freely [65]. Additionally, Zhu et al., (2010) suggested that pore sizes greater than 100 μm in 3D scaffolds are necessary to ensure nutrient diffusion throughout the scaffold, not just in superficial layers [66].

The SEM micrographs show U87-MG cell adhesion to the alginate–gelatin hydrogel scaffold structures (Figure 6d), as well as collapsed U87-MG spheroids in the alginate–gelatin hydrogel scaffold structures (Figure 6e). Moreover, 8 of the 24 known human integrin heteromers bind to the RGD recognition sequence, each responding differently to various ECM proteins containing RGD motifs [67]. As mentioned beforehand, gelatin contains the RGD sequence for cell adhesion and ensures mechanical support to the final matrix [56]. The primary RGD recognition motif mainly binds to α5β1 and αvβ3 integrins. Studies have suggested that changes in material composition and cross-linking can modify the availability of cell recognition sites, affecting binding [68]. Furthermore, αvβ3 integrin expression has been observed on human glioblastoma and U87-MG cells, with its expression potentially induced by chemical stress [69]. Many features of 3D spheroids such as their geometry, cellular organization, and polarity influence cellular function, influence responses to external stimuli, differentiation, gene expression, proliferation, and survival [24,30]. The 3D spheroids can hold a cellular organization and polarity, like those TMEs found in solid tumors, bearing a heterogenous cellular subpopulation within the 3D spheroid, including the actively proliferating, quiescent, hypoxic, and necrotic cells, and offering distinctive cell proliferation zones, namely, the proliferating zone, the quiescent viable zone, and the necrotic/hypoxic core [24,28,31,33].

### 2.7. Embedded Spheroids Within the Alginate–Gelatin Hydrogel Scaffold Exhibited Heterogenous Proliferating Cells, Neighboring Pseudopalisading Cells near the Center and a Necrotic Central Core

**Figure 7:**
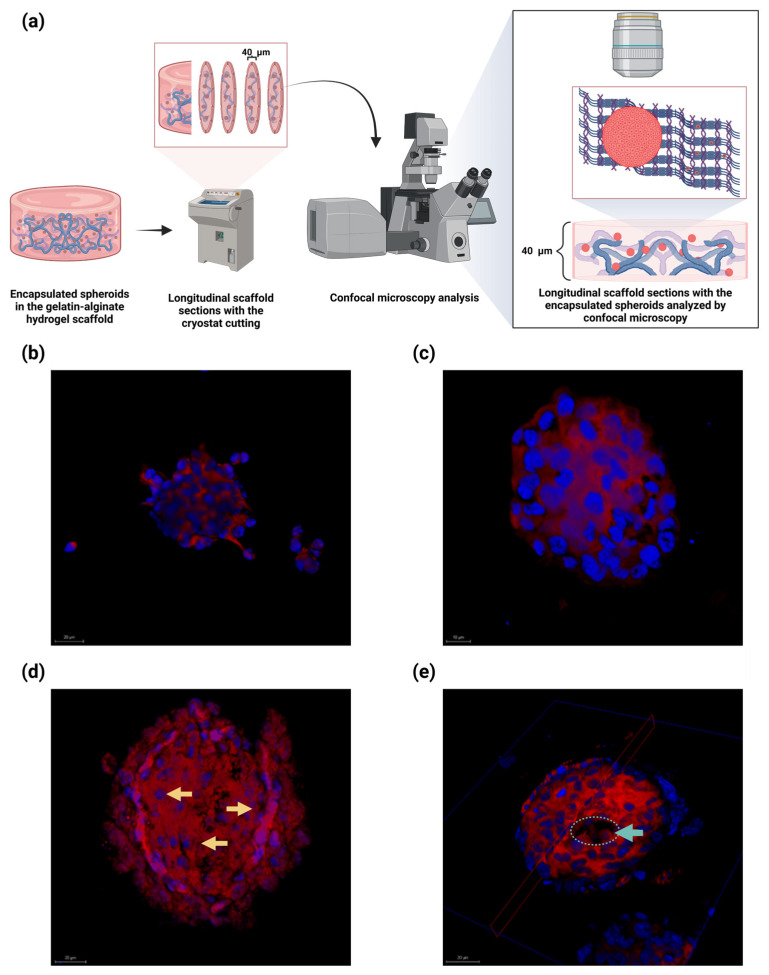
Confocal microscopy of the encapsulated U87-MG spheroids within the alginate–gelatin scaffolds analyzed at distinct days. (**a**) Schematic representation of sample processing. (**b**–**e**) Confocal micrograph of encapsulated spheroids within the alginate–gelatin scaffold at distinct days: (**b**) Day 1; (**c**) Day 3; (**d**) Day 5; (**e**) Day 10. Pseudopalisade cells (yellow arrows) and absence of central cells (cyan circle and arrow) are indicated. Nucleus staining with Hoechst 33,258 (blue) and membrane with rhodamine B (red). Scale bar, 20 µm. Confocal micrograph magnification: 40×. All experiments were conducted in triplicate.

To assess the cellular composition of the encapsulated U87-MG spheroids in the alginate–gelatin hydrogel scaffolds, we incubated them within the scaffolds for up to 10 days. The schematic representation of the subsequent sample processing is depicted (Figure 7a). Next, the different samples were analyzed by confocal microscopy (Figure 7b–e).

The U87-MG cells are detached from the spheroid on Day 1, possibly due to the highly invasive nature of the tumor cells (Figure 7b). An intact spheroid with highly delimited cell nuclei is observed on Day 5 (Figure 7c). A collapsed spheroid with a pseudopalisade cell arrangement is observed (yellow arrows) on Day 5, a distinctive feature like the histological features observed in GBM patients (Figure 7d). Remarkably, the U87-MG spheroid exhibited a lack of cells at its center on Day 10, suggesting spheroid central necrosis owing to oxygen and nutrient deprivation (Figure 7e). Our findings imply that the encapsulated U87-MG spheroids within the alginate–gelatin scaffolds developed heterogenous cell populations of outer proliferating cells, neighboring pseudopalisade cells near the center, and a necrotic central core, in a time-dependent manner.

We successfully assessed the embedded U87-MG spheroids in the alginate–gelatin hydrogel scaffold for different times for up to 10 days (Figure 7). We observed a pseudopalisade cell arrangement in the encapsulated U87-MG spheroids on Day 3, a distinctive histological hallmark of GBM (Figure 7c). Spheroid integrity, with a highly delimited cell nuclei, was also observed on Day 5 (Figure 7d). Notably, on Day 10, the encapsulated U87-MG spheroids within the hydrogel scaffold exhibited the absence of cells at its center on Day 10, suggesting spheroid central necrosis due to oxygen and nutrient deprivation (Figure 7e).

### 2.8. Embedded Spheroids in the Alginate–Gelatin Hydrogel Scaffold Expressed HIF-1α in a Time-Dependent Manner

**Figure 8:**
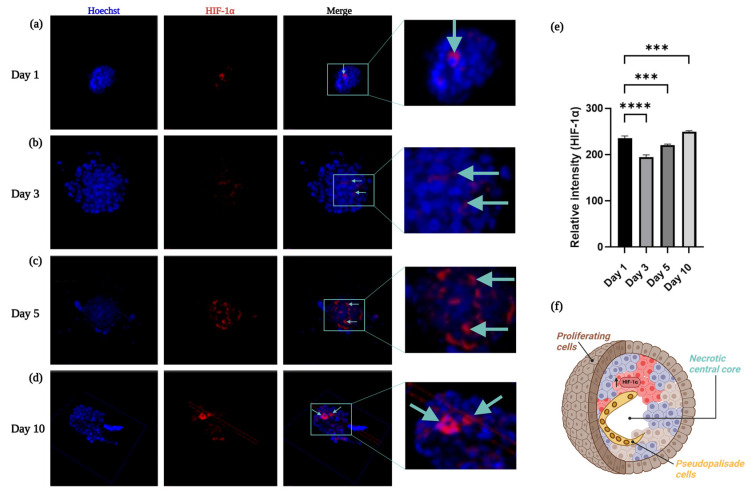
Immunofluorescence evaluation of HIF-1α. (**a**–**d**) HIF-1α expression in the encapsulated spheroids within the alginate–gelatin scaffold was evaluated at distinct days: (**a**) Day 1; (**b**) Day 3; (**c**) Day 5; (**d**) Day 10. The cyan arrows indicates HIF-1α detection. (**e**) Relative intensity of HIF-1α was estimated at distinct days. Nucleus staining with Hoechst 33,258 (blue) and HIF-1α detected with secondary antibody labeled with Alexa 594 fluorophore signal (red). All experiments were conducted in triplicate. *** *p* < 0.05; **** *p* < 0.05 (**f**) Schematic representation of the heterogenous cell populations of the outer proliferating cells (brown cells), neighboring pseudopalisade cells near the center (yellow cells), and a necrotic central core (absence of cells).

To verify the neighboring pseudopalisade cells near the center and the necrotic central core observations of the encapsulated spheroids in the alginate–gelatin hydrogel scaffolds, we assessed HIF-1α expression in the spheroids via an anti-HIF-1α mouse monoclonal antibody (Figure 8a–d) and a secondary antibody labeled with Alexa 594 fluorophore, which allowed for immunofluorescence and quantification of the relative intensity of HIF-1α through confocal microscopy and subsequent analyses (Figure 8e). A schematic representation of the heterogenous cell populations in the U87-MG spheroid is depicted (Figure 8f). 

HIF-1α expression varies among glioma grade, while HIF-1α expression is mainly located in the cytosol in low grade gliomas. In glioblastoma, HIF-1α immunoreactivity is distinctively strong in the neighboring areas of necrosis, mostly found in the nuclei [4,13,17]. This was consistent with our findings, except for Day 1, we observed an increase of HIF-1α expression in the U87-MG spheroids in the neighboring areas of necrosis, in a time-dependent manner, further validating our 3D hypoxia model. A previous study reported the generation of a uniform population of the U87-MG spheroids with an average diameter of 200 µm through the ultralow attachment (ULA) 96-well plate method. Moreover, these spheroids showed significantly higher expression of glioma stem cell (GSC) markers, such as hypoxia-inducible factor-1α (HIF-1α) and CD133 at 72 h, compared to GBM cells cultured in a monolayer [70]. We did not assess the expression of other hypoxia-related stem cell markers, such as CD133, OCT4, and SOX2 [63]. However, we consider them to be key hypoxia-related markers that must be considered in the future. Moreover, gene EGFR overexpression has been observed in 50% of GBM, and the prevailing EGFR mutation, EGFRvIII, was found in nearly 25–33% of all GBM patients [71]. As such, the oncogenic variant EGFRvIII is only found in malignant GBM cells and represents a standard target for GBM therapeutic development, retaining the potential for specificity in combination with efficacy and safety [72].

## 3. Conclusions

Glioblastoma (GBM) is a highly aggressive brain tumor characterized by poor prognosis and resistance to treatment. A hallmark of GBM is hypoxia, which triggers cellular responses, like microvascular proliferation and the expression of hypoxia-inducible factor 1 (HIF-1α), contributing to tumor progression and chemoresistance. To study GBM more effectively, 3D cell culture models are essential, as they mimic the tumor microenvironment (TME) more accurately than traditional 2D cultures. However, current 3D systems face challenges in reproducibility and high throughput. This study introduces a cost-effective and reproducible 3D culture system using U87-MG glioma spheroids embedded in an alginate–gelatin hydrogel scaffold. The hydrogel scaffold was optimized to mimic the TME, supporting cell growth, migration, and hypoxia. The physicochemical properties of the scaffold, including pore size, water absorption, and biocompatibility, were thoroughly characterized. The scaffold’s microstructure, revealed by SEM, showed interconnected pores suitable for cell attachment and nutrient diffusion, crucial for replicating GBM characteristics, including cellular organization and the development of necrotic centers. Notably, the U87-MG spheroids encapsulated in this scaffold exhibited HIF-1α expression and pseudopalisade formation, a typical feature of GBM. The alginate–gelatin hydrogel scaffold proves to be a reliable and scalable model for studying GBM, offering a platform to test therapeutic approaches and improve our understanding of the role of hypoxia and HIF-1α in GBM progression and resistance.

The development of a gelatin–alginate hydrogel scaffold for 3D cell culture has shown significant promise for cancer research, particularly in studying glioblastoma (GBM), a highly aggressive and malignant brain tumor. The hydrogel scaffold described here demonstrated reproducibility and consistency in its physicochemical characteristics, including its ability to support the growth and migration of the U87-MG glioblastoma cells. The 3D culture model closely mimicked the tumor microenvironment (TME), providing a more physiologically relevant system compared to traditional 2D cultures. Notably, the embedded U87-MG spheroids displayed characteristics of GBM, such as hypoxia and HIF-1α expression, which are crucial for understanding tumor progression and chemoresistance. The successful integration of gelatin and alginate in the scaffold enabled cell adhesion and migration, promoting the formation of spheroids that exhibited distinct histological features, like pseudopalisades and necrosis at the core, mimicking the behavior of GBM tumors in vivo. This model offers a reliable and cost-effective tool for studying the complex dynamics of glioblastoma, testing drug responses, and investigating potential therapies, while addressing the limitations of current 3D cell culture systems that can be time-consuming and lack reproducibility.

We successfully characterized and validated the alginate–gelatin hydrogel scaffold as an advantageous system to evaluate pathophysiological phenomena, like hypoxia and the correlation with latency and solid tumor metastatic cells, and the possibility to evaluate the penetration of novel compounds. Based on our previous research, we consider that the alginate–gelatin hydrogel scaffold developed in this study could represent a valuable resource for the forthcoming glioblastoma targeted EGFRvIII therapies, including immunotherapeutic-like conventional antibodies or VNAR single domain therapeutic carriers [73], as our hydrogel scaffold micrometric attributes emulate relevant GBM tumor features, including hypoxia, which could also prompt chemoresistance and radioresistance through HIF-1α expression.

Our findings also suggest that this hydrogel scaffold could be adapted for other cancer types, such as breast cancer and fibrosis, where the TME plays a crucial role in disease progression. The ability to control and manipulate specific features of TME, like hypoxia, opens new avenues for studying disease mechanisms and evaluating targeted therapies. This approach holds promise for improving preclinical cancer models and advancing therapeutic development. Future research can further refine these models by exploring other key markers and molecular pathways involved in cancer and GBM progression, offering a deeper understanding of tumor biology and treatment responses.

## 4. Materials and Methods

### 4.1. Generation of the Alginate–Gelatin Scaffold

#### 4.1.1. Hydrogel Scaffold Composition and Crosslinking

To mimic the brain’s extracellular matrix, rich in proteoglycans and hyaluronic acid, alginate (a linear polysaccharide) and gelatin with inactive type I collagen were used for hydrogel creation. To formulate the scaffolds, a range of sodium alginate (A1112, Sigma-Aldrich, St. Louis, MO, USA) concentrations of 1–10% (*w*/*v*) in Hank’s solution was tested and sterilized by autoclaving, aiming for a homogeneous solution. This alginate solution range was determined as ideal, showing low viscosity for easy material incorporation. Gelatin sheets from pork skin (bloom 230) were sterilized by UV at 320 nm during 15 min and dissolved in high-glucose DMEM (D5648, Sigma-Aldrich, Manassas, VA, USA)). Then combined with the alginate solution (1–10% range). The mixture was homogenized with mild agitation and placed in medical silicone molds (1 cm diameter × 0.5 cm height) at 4–8 °C for 1 h. The crosslinking was achieved by immersing the scaffolds in a 150 mM CaCl_2_ solution for 5–30 min, washed with PBS and sterile water at least five times each. Calcium chloride (CaCl_2_) is used to cross-link alginate in the alginate–gelatin hydrogels, which form a gel when it encounters the alginate. This crosslinking process is ionic, meaning that calcium ions bond with the carboxylate ions in the alginate [60,74]. Gelatin (food grade, 230 bloom) was incorporated from a 20% solution, and alginate was added in 2–5% (*w*/*v*) concentrations. The most stable scaffolds were those with a 3.75% alginate and 2.5% gelatin ratio, crosslinked for 30 min in CaCl_2_ at 150 mM. As this alginate–gelatin ratio demonstrated higher stability at 37 °C, this was employed for the subsequent experiments.

#### 4.1.2. Hydrogel Scaffold Weight Evaluation

The hydrogels (11.7 mm ± 0.2 and a height of 7.2 mm ± 0.3) kept in the 150 mM CaCl_2_ solution were stored at 4 °C before use. The excessive solution on the surface was wiped off with a piece of Kimwipe. Then, the hydrogel scaffolds were weighed in polystyrene containers in analytical balance (Precisa, Series 321, Dietikon, Switzerland), and their weight was measured over 96 h after incubation in Hank’s solution at 37 °C. The results show as a median of three measurements with nine replicates each.

#### 4.1.3. Fourier Transform Infrared (ATR-FTIR) Characterization

ATR-FTIR spectra were used to characterize the specific presence of chemical groups in the collagen–gelatin hydrogel to support the efficacy of the crosslinking procedure. FTIR spectra was obtained using a PerkinElmer Spectrum GX (Shelton, CT, USA) spectrometer with a 500 to 5000 cm^−1^ range, with KBr granules over 32 scans acquired at 4 cm^−1^ resolution with the subtraction of KBr background.

#### 4.1.4. Water Absorption Rate Determination

The water absorption rate was determined by direct immersion of the scaffold in sterile distilled water. The scaffold initial weight was determined before immersion (W_0_) at room temperature (25 °C). After immersion, the scaffold weight was determined at different times points (W_1_ to W_final_). The difference in water absorption was expressed as a percentage of weight change compared to W_0_. The assay was performed in triplicate.

### 4.2. Cell Culture in Monolayer and Spheroid with or Without Hydrogel Scaffold

#### 4.2.1. Cell Culture in Monolayer

The U87-MG cells (HTB-14, ATCC, Manassas, VA, USA) were thawed and cultured in EMEM medium (M4526, Sigma-Aldrich, Manassas, VA, USA)) supplemented with 10% FBS (fetal bovine serum, 41639, Sigma-Aldrich, Manassas, VA, USA). Cells were expanded in culture flasks until they reached 80% confluence, using trypsin-EDTA (T4174, Sigma-Aldrich, Manassas, VA, USA) for detachment after 5 min of incubation. After centrifugation, cells were resuspended in a maintenance medium (high glucose EMEM and 5% FBS), staining with trypan blue (T6146, Sigma-Aldrich, Manassas, VA, USA), counted with a Neubauer chamber, and their viability assessed.

#### 4.2.2. Spheroid Generation

Spheroids were generated using the suspension culture technique in low-adherence 12 wells plate. The U87-MG cells (500,000 cells per well) were seeded with 800 µL of high glucose DMEM and 5% FBS (as supplemented medium). The suspended spheroids were incubated in a shaking incubator (90 rpm, 37 °C, 5% CO_2_, 80% humidity, Infors Minitron CO_2_, (RG-MP-59, Bottmingen, CH-BL, Switzerland) for 3 days without medium change. Spheroid size was analyzed using inverted light microscopy and Leica LASX software (LASX Office 1.4.7 28921).

#### 4.2.3. Spheroid Cell Counting

To determine the number of cells that form each spheroid, suspended spheroids were aspirated individually, treated with trypsin-EDTA for 10 min, and disintegrated using a micropipette. After neutralizing the trypsin with a DMEM supplemented medium (5 mL), cells were counted using 1% trypan blue in a Neubauer chamber. The assay was performed in triplicate. 

#### 4.2.4. Cell Culture Within Scaffolds

Using the protocol described in Section 4.2.2, we generated spheroids then transferred and integrated them into an alginate–gelatin mixture (Section 4.1.1) by gently beating in clockwise circles five times with a sterile micropipette tip and evenly distributed within the scaffold, then incubated for 24 h under standard conditions. Then, cell viability of spheroid embedded in the hydrogel scaffold was assessed using resazurin reagent (R6892, Sigma-Aldrich, Manassas, VA, USA). The optimal incubation time for resazurin reduction was determined after 24 h of incubation, and readings at 570 nm and 600 nm in a Mark microplate spectrophotometer (10013301X, BioRad, Hercules, CA, USA). After this, scaffolds were incubated with new resazurin solution for 24 h, and absorbance was measured for 10 days, with samples taken on Days 1, 3, 5, and 10 after spheroid encapsulation. For the negative control, we used scaffolds without cells. The assay was performed in triplicate.

### 4.3. Embedded Spheroids in Alginate–Gelatin Scaffolds Microscopic Analysis

#### 4.3.1. Scaffold SEM Analysis with and Without Cells

Scaffold morphology was analyzed by scanning electron microscopy (SEM) after cryo-fracturing with nitrogen. Samples were coated with carbon and analyzed using a Tescan Mira3 LMU microscope (Brno, Czech Republic) at magnifications between 61× and 5000×. To analyze cell adhesion, we used the same protocol with embedded spheroid in the scaffold previously incubated for 3 days.

#### 4.3.2. Cell Staining and Confocal Microscopy

After 3 days of incubation, spheroids were encapsulated in scaffolds and incubated for 1, 3, 5, and 10 days at 37 °C with 5% CO_2_. Scaffolds were fixed in 4% paraformaldehyde, cryoprotected in 20% sucrose solution for 72 h, and sectioned using a cryostat (40 µm).

Cell nucleus and membrane staining were performed on samples on super frost slides (22037246, Thermo Fisher Scientific, Waltham, MA, USA). Nuclei were stained with Hoechst 33,258 (94403, Sigma-Aldrich, Manassas, VA, USA) at 0.2 µg/mL in 1× PBS for 15 min, followed by three 1× PBS washes. Membranes were stained with Rhodamine B (234141, Sigma-Aldrich, Manassas, VA, USA) at 1.5 mg/mL in absolute ethanol for 5 min, followed by three washes with 1× PBS. Stained slides were observed under a Leica TCS SPE confocal microscope (DM5500B-CS, Leica, Wetzlar, DEU) at 20×, 40×, and 60× magnification, and images were acquired and analyzed using LAS X software (version v3.4.218368) with excitation and emission wavelengths for FITC, Rhodamine, and Hoechst.

### 4.4. HIF1-α Analysis of Spheroids in the Alginate–Gelatin Scaffolds

#### HIF1-α Immunofluorescence Analysis

The nucleus was stained with Hoechst 33,258 and the membrane with rhodamine B, followed by incubation with anti-HIF-1α mouse monoclonal antibody (sc-13515, Santa Cruz Biotechnology, Dallas, TX, USA) at 2 µg/mL for 1 h at 37 °C. The secondary antibody labeled with anti-HIF Alexa 594 (A-21125, Thermo Fisher Scientific, Waltham, MA, USA) was added at 1 µg/mL, and the slides were sealed with a coverslip.

Relative fluorescence intensity of the Alexa 594 fluorophore was analyzed using ImageJ 1.x (version 1.53c) and FIJI software version 2.16.0 [75]. Images were processed, converted to binary, and analyzed for particle quantification. Results were plotted, and statistical analysis was performed using GraphPad Prism (version 10.4.1). The assay was performed in triplicate.

## Data Availability

The original contributions presented in this study are included in the article. Further inquiries can be directed at the corresponding author.
